# Health status in subjects with suspected obstructive sleep apnea and comparison with a general population

**DOI:** 10.1038/s41598-018-23904-3

**Published:** 2018-04-03

**Authors:** Kornelia K. Beiske, Knut Stavem

**Affiliations:** 1 Department of Neurology and Clinical Neurophysiology, Medical Division, Akershus University Hospital, Lørenskog, Norway; 2 Institute of Clinical Medicine, Campus Akershus University Hospital, University of Oslo, Oslo, Norway; 3 Department of Pulmonary Medicine, Akershus University Hospital, Lørenskog, Norway; 4 Health Services Research Unit, Akershus University Hospital, Lørenskog, Norway

## Abstract

The purpose of this study was to assess health status (HS) in patients with clinical suspicion of obstructive sleep apnea (OSA) in order to estimate the dose response relationship between HS and OSA severity, and to compare HS in this clinical cohort with a general population sample (N = 5000). Patients referred to an overnight sleep study due to suspected OSA, whom also responded to the SF-36 questionnaire, were included (N = 418). Of these, 194 showed normal findings, while 111, 60 and 53 demonstrated mild, moderate and severe OSA, respectively. Mean age was 47.5 (SD 11.9) and 69% were males. Only the mental health scale (p = 0.015) and mental component summary score (p = 0.023) were associated with OSA severity. This association, however, disappeared in multivariable analysis. All SF-36 scores in the sleep study group were lower than that of the general population sample, in both unadjusted and multivariable linear regression analysis. In this study, there was a lack of association between OSA severity and general HS. However, as a whole, patients in this clinical population referred to an overnight sleep study due to suspected OSA had impaired HS on all scales compared to a general population, with greatest differences in the vitality domain.

## Introduction

Obstructive sleep apnea (OSA) is a common disorder characterized by recurrent episodes of complete and partial airway obstruction, which may result in an increased risk of cardiovascular diseases and metabolic disorders and subsequently have an adverse effect on quality of life^1^. Recent studies show increasing prevalence of OSA in the general adult population with findings of moderate OSA from 6 to 23% for adult women and from 13 to 50% for adult men in the general population^2,3^.

During the last decades, quality of life assessments in OSA patients have become a main focus in statements on sleep apnea published by The American Thoracic Society^4^ and the American Sleep Disorders Association^5^, leading to increased use of health status measures in both the clinical and research setting. Health status (HS) is a multidimensional concept that usually includes self-report of the way in which physical, mental, social, or other domains of well-being are affected by a disease or its treatment^6^. There is no absolute consensus for the definition of HS. This results in interchangeable use of the terms health-related quality of life (HRQoL) and HS, as both describe self-assessment of the aforementioned domains^7^. One of the most widely used questionnaires for the assessment of general HS is the SF-36, which is used to compare general and specific populations, assess the relative burden of disease, and differentiate health benefits according to treatments^8^.

Several studies have found that HS, as assessed by the SF-36, in untreated OSA patients is significantly impaired in numerous domains compared to general population samples^9–11^. Higher apnea-hypopnea index (AHI) has been in a dose-response fashion related to lower scores on six of eight SF-36 domains (mental health, social functioning, role physical, physical functioning, vitality and general health) in a large population based sample^12^. Males with mild OSA have shown poorer scores for the domains of role emotional, role physical, vitality and mental health when compared to patients with normal findings^13^. In another clinical population sample, patients with mild and moderate OSA scored lower in the domains of physical functioning and role physical, and patients with moderate to severe OSA scored lower on vitality compared to patients without respiratory disturbances^14^. In contrast, others have found no significant associations between AHI and HS in clinical populations^15–17^. Sleep quality has been shown to broadly influence general HS as assessed by the SF-36^18^, and the vitality domain has also shown associations with sleep disruption in OSA patients^19^.

The estimated prevalence of OSA in middle-aged adults in the general Norwegian population is approximately 25%^20^, but there is limited knowledge of the HS of persons with OSA in Norway^21^. The objective of this study was to use the SF-36 to: (1) estimate the dose response relationship between HS and OSA severity, grouped as mild, moderate or severe OSA, and (2) to compare HS in this clinical cohort with a general population sample.

## Methods

### Participants

#### Sleep study group

**Figure 1:**
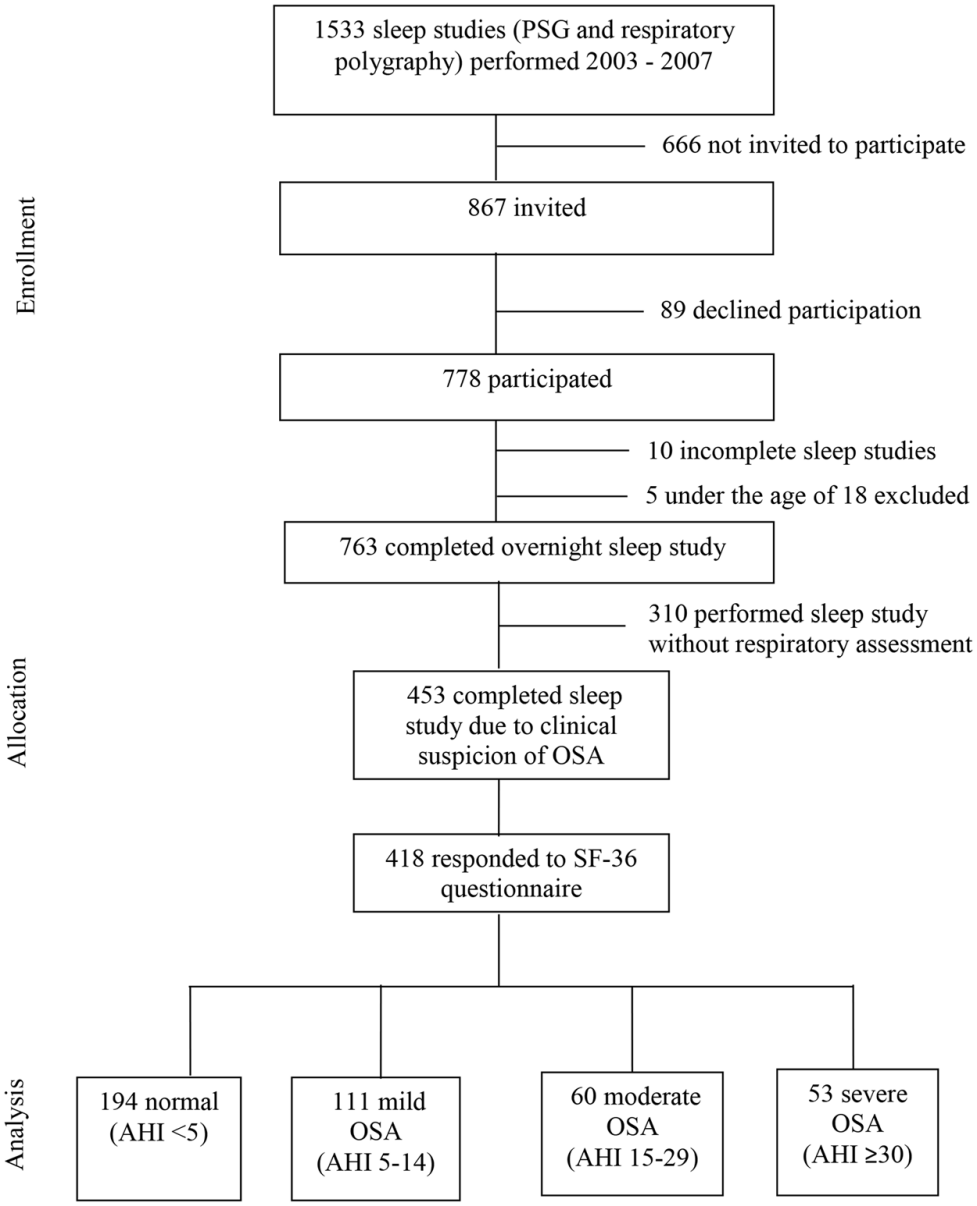
Study population flow chart.

This cross-sectional study wanted to include all subjects referred to the Department of Clinical Neurophysiology, Akershus University Hospital between October 2003 and December 2007 for a sleep study due to clinical suspicion of OSA. Referrals for the majority of patients came from general practitioners, neurologists or ear-nose-throat specialists. The inclusion process was delegated to neurophysiology technicians at the Department of Clinical Neurophysiology. During this period 1533 sleep studies were performed, 89 subjects declined to participate and 666 were not invited for unknown reasons, resulting in a total of 755 non-participants, and an inclusion rate of 51%. During this inclusion period, PSGs without respiratory assessments were also performed for patients who did not have clinical findings consistent with sleep apnea. These patients were not included in this study. In total, 418 subjects with clinically suspect OSA complaints who also fully completed the SF-36 questionnaire were included for further analysis (Figure 1).

The Regional Committee for Medical Research Ethics, Helse-Sør-Øst approved the project’s ethical aspects (210-03096). Informed, written consent was obtained from all participants included in the study.

#### General population

General population values were collected from a survey conducted as part of the Norwegian Lifestyle Survey by Statistics Norway in 2002^22^. The survey comprised 9698 members of the general population ≥16 years of age that were representative for Norway. The study included home and telephone interviews prior to a postal survey. The postal questionnaire included the Norwegian SF-36 version 1.2^23^ mailed between 15 November 2002 and 15 May 2003. We had access to data collected from persons that responded to both postal survey and interview. Of these, 5000 also responded to all SF-36 questionnaire domains, and were used in further analysis.

### Questionnaire

Participants in the sleep study group filled out a questionnaire that included items about demographics, smoking status, presence of comorbid conditions (high blood pressure, diabetes mellitus and anxiety or depression), a Norwegian version of the Epworth sleepiness scale (ESS)^24^, and a Norwegian version of the standard SF-36 questionnaire, version 1.2^23^.

The SF-36 is a general HS questionnaire. It assesses eight dimensions of health including physical functioning, role limitations related to physical problems, bodily pain, general health perception, vitality, social functioning, role limitations due to emotional problems and mental health. Raw scores are converted to a scale from 0 to 100. Higher transformed scores indicate better HS according to the SF-36 scoring manual^8^. The scales are also aggregated to provide two summary scales, the physical component summary (PCS) and mental component summary (MCS), which are scored on a standardized scale designed for comparison with a US general population with mean 50 (SD 10)^25^.

### Sleep studies

Participants in the sleep study group who were evaluated only for sleep apnea underwent an ambulant PG (n = 343) using the Embla system (EMBLA; Medcare-Flaga; Reykjavik, Iceland), recording airflow with thermistors, oxygen saturation by finger-pulse oximeter, heart rate, abdominal and thoracic respiratory movements, body position and snoring. Participants with symptoms suspect for both OSA and other sleep disorders were referred to a polysomnography (PSG) (n = 75) using the same ambulant Embla system, but with additional surface electrode recordings of the electroencephalogram (C3-A2, C4-A1, O1-A2, O2-A1), electrooculograms (EOG1 and EOG2), electrocardiogram, as well as *m. submentalis* and bilateral *m. tibialis anterior* electromyograms. The sleep studies were scored according to the criteria of the American Academy of Sleep Medicine Task Force by physicians specializing in clinical neurophysiology^26^.

### Statistical analysis

Descriptive statistics were presented using means with SDs or number (%). The characteristics of participants in the sleep study group and the general population sample were compared using the *t*-test for continuous and chi-square test for discrete variables.

SF-36 scale scores were computed according to standardized criteria^8,25^. We compared SF-36 scale scores when participants were grouped according to OSA severity (normal, mild, moderate, severe) using the Kruskal-Wallis test, and the Wilcoxon-Mann-Whitney test when comparing participants in the sleep study group with the general population sample.

In further analysis, we focused on the SF-36 domains of physical functioning and vitality, which in previous studies have been associated with sleep disorders and therefore expected to be most sensitive to symptoms of OSA^10,12,27^, as well as the two component summary scores, PCS and MCS. We chose to adjust the comparisons of HS for the variables age, gender, education, body mass index (BMI), ESS > 10 and comorbid conditions, which in previous studies have shown associations between OSA and quality of life^10,12,14,27,28^, where available.

In the sleep study group we performed multivariable linear regression analysis for the SF-36 domains physical functioning, vitality, PCS and MCS adjusting for age per 10 years, gender, education level, BMI as well as ESS ≤ 10 or >10, presence of high blood pressure, diabetes mellitus and anxiety or depression for participants grouped according to OSA severity compared to those with AHI < 5 (normal). All independent variables were forced into the models.

When comparing participants in the sleep study group to the general population sample using multivariable linear regression analysis and the same dependent variables as above, we adjusted for age per 10 years, gender, education level, and BMI, again forcing all independent variables into the models.

Some of the residuals in the multivariable linear regression models did not comply with a normal distribution, but log-transformation or square-root transformation of the affected dependent variables did not resolve this situation. We therefore used the untransformed values for the dependent variables, but used bootstrapped 95% confidence intervals with 500 replications in all models. Missing values were not imputed.

We chose a 5% significance level, using two-sided tests. The Stata version 14.1 (StataCorp, College Station, TX, USA) was used for analyses.

### Data availability statement

The patient data analysed during the current study are not publicly available due to ethical and legal constraints, but anonymized data are available from the corresponding author on reasonable request.

The general population data that support the findings of this study are available from Norwegian Centre for Research Data (NSD) but restrictions apply to the availability of these data, which were used under license for the current study, and so are not publicly available. Data are however available from the authors upon reasonable request and with permission of NSD.

### Ethical approval

All procedures performed in studies involving human participants were in accordance with the ethical standards of the institutional and/or national research committee and with the 1964 Helsinki declaration and its later amendments or comparable ethical standards.

### Informed consent

Informed consent was obtained from all individual participants included in the study.

## Results

### Descriptive statistics for the sleep study group

Participants in the sleep study group comprised more males (p < 0.001), had higher BMI (p < 0.001), a lower percentage of smokers (p = 0.044) and a greater percentage of participants with university or college degree than the general population sample (p < 0.001), but there was no difference in age (p = 0.37) between the groups. There was increasing BMI with increasing OSA severity, and participants with severe OSA had the highest percentage of ESS > 10 (Table 1).

**Table 1:** Descriptive statistics for patients referred to an overnight sleep study due to a clinical suspicion of obstructive sleep apnea (sleep study group) and general population sample who responded to all SF-36 questionnaire domains.

	Normal	Obstructive sleep apnea			Total	General population	p^**^
		Mild	Moderate	Severe	418	5000	
AHI^*^	<5	5–14	15–29	≥30			
*N*	194	111	60	53			
	Mean (SD) or N (%)^**^	Mean (SD) or N (%)^**^	Mean (SD) or N (%)^**^	Mean (SD) or N (%)^**^	Mean (SD) or N (%)^**^	Mean (SD) or N (%)^**^	
Age, years	43.7 (12.1)	49.7 (11.2)	52.2 (9.9)	50.8 (10.1)	47.4 (11.9)	46.7 (16.5)	0.37
Male gender	105 (54)	82 (74)	50 (83)	49 (92)	286 (68)	2435 (49)	<0.001
Body mass index, kg/m²	27.4 (5.1)^1^	28.7 (4.8)	29.8 (4.5)	32.7 (5.9)	28.8 (5.3)	24.8 (5.1)^2^	<0.001
Education							<0.001
Schooling up to 10 years	34 (18)	28 (25)	14 (24)	13 (25)	89 (22)	659 (13)	
Schooling 11–13 years	76 (40)	42 (38)	20 (34)	20 (39)	158 (38)	2816 (57)	
University or college degree	81 (42)	41 (37)	25 (42)	18 (35)	165 (40)	1471 (30)	
Current smoker	63 (33)^1^	31 (28)	17 (28)	20 (38)^3^	131 (32)	1825 (37)^4^	0.044
Epworth sleepiness scale >10	87 (47)^5^	42 (42)^6^	24 (44)^7^	28 (55)^8^	181 (46)		
High blood pressure	17 (11)^9^	14 (15)^10^	12 (25)^11^	12 (30)^12^	55 (17)		
Diabetes mellitus	8 (5)^9^	8 (9)^10^	4 (8)^11^	5 (13)^12^	25 (8)		
Anxiety or depression	34 (23)^9^	10 (11)^10^	13 (27)^11^	7 (18)^12^	64 (19)		

^*^Apnea-hypopnea index.

^**^Continuous variables are presented using mean (SD), and categorical variables using number (percent).

^***^Comparison of total sleep group and general population using the *t*-test for continuous and Chi-square test for discrete variables.

^1^N = 192.

^2^N = 4926.

^3^N = 52.

^4^N = 4998.

^5^N = 187.

^6^N = 101.

^7^N = 54.

^8^N = 51.

^9^N = 151.

^10^N = 91.

^11^N = 48.

^12^N = 40.

There was no difference in age when comparing participants (N = 778) to non-participants (N = 755), with mean (SD) age of 45.1 (12.9) and 45.8 (16.0), respectively (p = 0.38). There was a similar distribution of gender among participants and non-participants, 62% and 59% males respectively (p = 0.37).

### Dose-response between health status and OSA severity

Comparison of unadjusted SF-36 scale scores between participants in the sleep study group categorized according to OSA severity showed higher values for participants with mild, moderate and severe OSA on the mental health and MCS scales than those with normal findings (AHI < 5) (Table 2). Higher transformed SF-36 scale scores indicate better HS.

**Table 2:** SF-36 scale scores in patients referred to an overnight sleep study due to a clinical suspicion of obstructive sleep apnea (sleep study group; N = 418) categorized according to OSA findings.

	Normal	Obstructive sleep apnea			*P* ^***^
		Mild	Moderate	Severe	
*AHI* ^*^	<5	5–14	15–29	≥30	
N	194	111	60	53	
SF-36 scales^**^	Mean (SD)	Mean (SD)	Mean (SD)	Mean (SD)	
Physical functioning	81.4 (19.6)	79.9 (20.0)	79.5 (21.1)	76.7 (16.9)	0.052
Role physical	54.5 (41.0)	53.8 (41.0)	51.7 (41.4)	51.9 (39.8)	0.92
Bodily pain	60.8 (28.2)	62.6 (26.8)	63.1 (29.9)	59.7 (30.2)	0.92
General health	59.5 (25.0)	61.4 (23.4)	59.1 (24.2)	59.2 (21.7)	0.88
Vitality	34.8 (23.5)	40.4 (20.4)	39.7 (23.2)	37.6 (20.4)	0.118
Social functioning	69.1 (27.6)	76.0 (25.2)	71.0 (28.1)	75.9 (22.2)	0.143
Role emotional	67.4 (38.7)	67.9 (39.2)	70.0 (36.7)	73.6 (38.9)	0.68
Mental health	68.8 (18.8)	75.4 (15.9)	73.3 (17.9)	73.1 (19.2)	0.020
Physical component summary^****^	44.3 (10.8)	43.7 (10.6)	43.3 (10.6)	42.1 (9.7)	0.49
Mental component summary^****^	43.8 (11.0)	47.2 (10.4)	46.4 (10.6)	47.6 (11.0)	0.023

^*^Apnea-hypopnea index.

^**^Scored on a 0–100 scale.

^***^Kruskal-Wallis test.

^****^Standardized for comparison with a U.S. general population with mean 50 (SD 10).

There was no difference in overall HS between participants in the sleep study group categorized according to OSA severity and those in the normal group (AHI < 5) after adjustment for gender, education, age per 10 years, BMI, ESS > 10, presence of high blood pressure, diabetes mellitus and anxiety or depression. In this multivariable linear regression model, vitality and MCS scales were positively associated with age, but negatively with the presence of anxiety or depression. ESS score > 10 was negatively associated with the vitality scale, and BMI and the presence of diabetes mellitus were negatively associated with the physical functioning scale. The PCS scale was negatively associated with high blood pressure (Table 3).

**Table 3:** Determinants of health status in sleep study participants (sleep study group). Coefficients and 95% confidence intervals (95% CI) for SF-36 scales according to categories of obstructive sleep apnea severity. Multivariable linear regression adjusted for gender, education, age, body mass index, Epworth sleepiness scale score > 10 as well as presence of high blood pressure, diabetes and anxiety or depression.

	Physical functioning (*N* = 304)			Vitality (*N* = 304)			Physical component summary (*N* = 304)			Mental component summary (*N* = 304)		
	Coef.^*^	95% CI	*P*	Coef.^*^	95% CI	*P*	Coef.^*^	95% CI	*P*	Coef.^*^	95% CI	*P*
Obstructive sleep apnea												
Normal (AHI^**^ < 5)	0											
Mild (AHI^**^ 5–14)	−0.7	−5.1–3.7	0.76	−0.6	−6.1–4.8	0.82	−0.8	−3.6–2.1	0.60	0.1	−2.5–2.7	0.95
Moderate (AHI^**^ 15–29)	4.1	−1.2–9.4	0.126	3.4	−4.6–11.2	0.41	0.4	−3.5–4.4	0.83	1.4	−2.0–4.8	0.43
Severe (AHI^**^ > 30)	−0.1	−7.1–6.8	0.98	1.9	−8.1–9.0	0.92	−1.1	−5.1–2.9	0.59	1.4	−1.7–4.5	0.36
Female gender	−3.6	−8.3–1.0	0.128	−2.0	−7.3–3.2	0.45	−0.9	−3.8–1.9	0.53	−1.9	−4.5–0.67	0.147
Education												
Schooling up to 10 years	0											
Schooling 11–13 years	2.2	−3.7–8.1	0.47	1.9	−5.5–9.3	0.61	0.6	−3.0–4.2	0.74	0.3	−2.7–3.3	0.84
University or college degree	3.2	−2.8–9.2	0.29	1.9	−5.4–9.1	0.62	0.1	−3.3–3.5	0.94	−1.0	−3.8–1.8	0.48
Age per 10 years	−1.1	−3.0–0.7	0.24	4.4	2.1–6.6	<0.001	−0.3	−1.3–0.8	0.64	1.85	0.8–2.9	<0.001
Body mass index, kg/m^2^	−0.8	−1.3–−0.3	0.001	0.4	−0.1–1.0	0.122	−0.1	−0.4–0.1	0.31	0.2	−0.06–0.4	0.143
Epworth Sleepiness Scale > 10	−1.0	−5.0–3.0	0.63	−9.7	−14.3–−5.1	<0.001	−1.2	−3.7–1.3	0.34	−1.2	−3.1–0.8	0.25
High blood pressure	−5.2	−11.4–1.0	0.101	−2.0	−9.1–5.2	0.59	−3.8	−6.8–−0.7	0.016	0.3	−2.4–3.1	0.81
Diabetes mellitus	−9.9	−19.6–−0.3	0.043	−7.8	−16.9–1.4	0.098	−3.8	−8.9–1.2	0.135	−1.6	−5.6–2.5	0.45
Anxiety or depression	−4.1	−9.9–1.7	0.163	−12.1	−18.3–−5.8	<0.001	−1.3	−4.2–1.6	0.38	−12.7	−15.7–−9.6	<0.001

^*^Unstandardized beta coefficient.

^**^Apnea-hypopnea index.

### Comparison of health status between sleep study group and general population

Unadjusted SF-36 scores in the sleep study group were lower on all scales than in the general population sample (Table 4).

**Table 4:** Distribution of SF-36 scale scores in patients referred to an overnight sleep study due to a clinical suspicion of obstructive sleep apnea (sleep study group) compared to the general population sample.

*N*	Sleep study group	General population	*P* ^**^
	418	5000	
SF-36 scale^*^	Mean (SD)	Mean (SD)	
Physical functioning (PF)	80.1 (19.6)	87.1 (19.5)	<0.001
Role-physical (RP)	53.6 (40.8)	77.4 (36.9)	<0.001
Bodily pain (BP)	61.5 (28.3)	74.0 (25.6)	<0.001
General health (GH)	59.9 (24.0)	75.4 (21.7)	<0.001
Vitality (VT)	37.3 (22.4)	61.1 (20.4)	<0.001
Social functioning (SF)	72.1 (26.5)	86.8 (20.7)	<0.001
Role emotional (RE)	68.7 (38.5)	84.9 (31.0)	<0.001
Mental health (MH)	71.8 (18.1)	80.4 (15.4)	<0.001
Physical component summary^***^	43.7 (10.6)	49.4 (10.2)	<0.001
Mental component summary^***^	45.6 (10.9)	52.3 (9.0)	<0.001

^*^Scored on a 0–100 scale.

^**^Wilcoxon-Mann-Whitney test.

^***^Standardized for comparison with a U.S. general population with mean 50 (SD 10).

After adjustment for gender, education, age per 10 years and BMI, participants in the sleep study group scored lower than the general population on the physical functioning, vitality, PCS and MCS scales, with the largest difference found on the vitality scale (unstandardized beta coefficient of −23.1 (CI 95% −25.4 to −20.9) p < 0.001) (Table 5). All variables used for adjustment, except BMI for the MCS scale, showed associations with the assessed domains.

**Table 5:** Coefficients and 95% confidence intervals (95% CI) for SF-36 scales in participants with suspected obstructive sleep apnea (sleep study group; N = 410) compared to the general population sample (N = 4875).

	Physical functioning (*N* = 5285)			Vitality (*N* = 5285)			Physical component summary (*N* = 5285)			Mental component summary (*N* = 5285)		
	Coef.^*^	95% CI	*P*	Coef.^*^	95% CI	*P*	Coef.^*^	95% CI	*P*	Coef.^*^	95% CI	*P*
Cohort												
General population	0											
Clinical suspicion of OSA	−4.7	−6.7–−2.7	<0.001	−23.1	−25.4–−20.9	<0.001	−4.5	−5.6–−3.5	<0.001	−7.0	−8.1–−5.8	<0.001
Gender												
Female	−4.8	−5.7–−3.9	<0.001	−5.6	−6.7–−4.4	<0.001	−2.1	−2.6–−1.6	<0.001	−1.3	−1.8–−0.8	<0.001
Education												
Schooling up to 10 years	0											
Schooling 11–13 years	6.3	4.4–8.1	<0.001	3.6	1.7–5.5	<0.001	2.5	1.6–3.3	<0.001	1.8	0.9–2.6	<0.001
University or college degree	10.7	8.9–12.5	<0.001	5.7	3.7–7.7	<0.001	4.7	3.8–5.6	<0.001	2.3	1.4–3.2	<0.001
Age per 10 years	−4.4	−4.7–−4.0	<0.001	0.7	0.3–1.1	<0.001	−2.1	−2.2–−1.9	<0.001	0.6	0.5–0.8	<0.001
Body mass index, kg/m^2^	−0.7	−0.8–−0.5	<0.001	−0.4	−0.5–−0.2	<0.001	−0.3	−0.4–−0.3	<0.001	0.02	−0.05–−0.1	0.53

Multivariable linear regression adjusted for gender, education, age and body mass index.

^*^Unstandardized beta coefficient.

## Discussion

### Principal findings

In this study of patients with suspected OSA referred to an overnight sleep study, we could not show an overall difference in SF-36 scale scores according to OSA severity after adjusting for confounders. However, when comparing participants with OSA to those with normal findings, increasing age was associated with higher SF-36 scores for the domains of vitality and MCS, while the presence of anxiety or depression was associated with lower scores in these domains. ESS > 10 was also associated with lower vitality scale scores.

The lack of a dose response relationship between HS and OSA severity found in this study is in line with previous studies assessing OSA severity and HS^29,30^, although associations between OSA severity and certain SF-36 domains have also been found in both clinical and non-clinical populations^12–14^. In a population-based study, mild OSA was related to lower scores on the vitality scale, while more severe OSA was more broadly associated with reduced HS^10^.

Unadjusted analysis of the sleep study group showed that patients with OSA had higher SF-36 scores for both the mental health scale and MCS scores compared to patients with normal findings. This supports findings from other studies where patients with severe OSA reported higher mental health scale scores than normative SF-36 data^10^, and less depressive symptoms than patients with normal findings^31^. However, this finding is not universal across studies, as other studies have reported lower mental health scale scores for patients with OSA than normal controls^28,32^. These inconsistencies may be related to differences in sample composition, investigation methods, and varying adjustments of potential confounding variables such as age, BMI, and comorbid medical conditions.

All SF-36 scale scores in the sleep study group were lower compared to the general population sample, both unadjusted and when controlled for age, gender, education and BMI. The largest difference was found on the vitality scale, which may be explained by this domain’s responsiveness to sleep disruption^19^. Lower HS in OSA patients compared to general population samples in several SF-36 domains are previously documented^33,34^, with some of them also showing significant improvements in HS after treatment with continuous positive airway pressure (CPAP) therapy, with most obvious improvements in those with severe OSA^34,35^. However, the present study was a cross-sectional study, and none had CPAP therapy.

### Strengths of the study

The present study included a relatively large number of patients referred to an overnight sleep study with suspected OSA and could be compared to the SF-36 scores of a general population sample directly. Some previous studies assessing HS in OSA patients have similarly included consecutive patients referred to a sleep study^15,27^, while others have used a selection of OSA patients^9,18^ or investigated general population samples^10,12^. To our knowledge, there are no prior studies assessing general HS in a Norwegian clinical OSA population in relation to general population norms. Data from the general population sample used in this study was collected at about the same time and had similar age and smoking status as the sleep study group. Increasing age and smoking are both associated with OSA^36,37^.

### Limitations of the study

#### Participant selection

One main limitation of the present study was the recruitment process of participants. The intention of the study was to include all consecutive patients referred to an overnight sleep study. Recruitment to the study was difficult to accomplish during periods of the year and varied over time, possibly leading to a recruitment bias. This non-consecutive sampling may have resulted in a sampling bias, and consequently question the representativeness of the sample and generalizability of the findings. In spite of adjustment for important demographic characteristics, findings from this study, based on a selected clinical population referred to sleep study due to clinical symptoms of OSA, cannot fairly be compared with a general population sample. For example, the selected clinical population will naturally have a higher prevalence of OSA than a random general population sample.

#### Sleep study recordings and scoring

Another limitation is the difference in measurement methods of OSA. More than 80% of the sleep studies were performed using PG, which may underestimate the AHI compared to a PSG with both respiratory and EEG parameters. In addition, the recordings were performed using only a thermistor, which may have affected correct identification of respiratory events. According to latest scoring guidelines, a thermistor is recommended for identifying apneas and a nasal pressure transducer for identifying hypopneas^38^. The 1999 American Academy of Sleep Medicine (AASM) scoring rules used in this study are more liberal than current criteria, as they do not necessarily distinguish obstructive apneas from hypopneas, and do not require a minimum fall in oxygen desaturation or arousal in order to score respiratory events^26^. Thus, the chosen recording and scoring methods make comparison of results with studies using current AASM guidelines difficult.

#### Excessive daytime sleepiness and comorbidities

Comparison of ESS scores and presence of comorbidities between the sleep study group and general population would have been advantageous, as EDS and comorbid illnesses such as hypertension and depression are associated with lower HS in OSA patients^18,27^, but neither ESS scores nor comparable information regarding comorbidities were available for the general population sample.

### Clinical implications

Although OSA severity was not associated with impaired HS, patients in the sleep study group had lower HS than the general population sample. This indicates that the impairment in HS experienced by OSA patients may be caused by factors not necessarily related to OSA severity as currently defined by the AHI. For example, this may be related to sleep problems or reduced sleep quality in general, as sleep quality broadly influences general HS^18^, and the vitality domain is associated with sleep disruption in OSA patients^19^. Similarly, persons reporting poor sleep quality due to other sleep disorders, e.g. RLS and central disorders of hypersomnolence, also have lower general HS compared to general population samples^39,40^. For this reason, clinicians should also consider other sleep disorders as well as physical and mental conditions when assessing these patients.

The findings also serve as a reminder that the patients being referred for a sleep study are heterogeneous and present with a number of complaints or unspecific symptoms, which may lead to reduced HS independent of OSA or not. The unspecific nature of the symptoms makes screening for OSA difficult, and there is currently uncertainty about the accuracy or clinical utility of all potential screening tools^41^.

## Conclusion

The present study has affirmed the lack of association between OSA severity and general HS, as measured by the SF-36 instrument. In contrast, compared to a general population sample, the general HS of Norwegian patients referred to an overnight sleep study due to clinical suspicion of OSA was impaired, with greatest differences in the vitality domain. These findings suggest that this difference may not necessarily be due to the presence of OSA, but may reflect the unspecific nature of symptoms in OSA compared to other sleep disorders or other confounding factors.
